# Demographic and Family Network Influences on Psychosocial Health in Agincourt, South Africa: A SEM Analysis

**DOI:** 10.1093/geroni/igaf122.3138

**Published:** 2025-12-31

**Authors:** Lawrence Ugwu, JohnBosco Chika Chukwuorji, Erhabor S Idemudia

**Affiliations:** North-West University, Mafikeng, North-West, South Africa; Michigan State University, East Lansing, Michigan, United States; North-West University, Mafikeng, North-West, South Africa

## Abstract

Demographic determinants, family networks, and daily activity play critical roles in shaping the psychosocial health of older adults. In this study, we examined the predictive effects of age, gender, marital status, and family structure, as indicated by the number of children and grandchildren, including resident grandchildren, on subjective well-being (SWB), depression, and physical activity among older adults in Agincourt, South Africa. Our representative sample, with a mean age of 71.96 years (SD = 8.81) and nearly equal gender distribution (1,330 males and 1,445 females), was analysed using structural equation modelling. Results indicate that higher daily activity levels and robust family engagement, particularly having resident grandchildren, were significantly associated with reduced depression, enhanced physical activity, and improved SWB. Being currently married and maintaining active family interactions further strengthened these associations. These findings emphasise the importance of social integration and family support in mitigating mental health challenges and promoting physical activity among older adults. The study offers valuable insights for developing community-based interventions and policies to enhance the quality of life for ageing populations by reinforcing family networks and encouraging active engagement in daily life.

